# Probiotics *Bacillus licheniformis* Improves Intestinal Health of Subclinical Necrotic Enteritis-Challenged Broilers

**DOI:** 10.3389/fmicb.2021.623739

**Published:** 2021-05-18

**Authors:** Liugang Kan, Fangshen Guo, Yan Liu, Van Hieu Pham, Yuming Guo, Zhong Wang

**Affiliations:** State Key Laboratory of Animal Nutrition, College of Animal Science and Technology, China Agricultural University, Beijing, China

**Keywords:** subclinical necrotic enteritis, *Bacillus licheniformis*, intestinal health, immune response, microflora, broiler chicken

## Abstract

Necrotic enteritis infection poses a serious threat to poultry production, and there is an urgent need for searching effective antibiotic alternatives to control it with the global ban on in-feed antibiotics. This study was conducted to investigate the effects of dietary *Bacillus licheniformis* replacing enramycin on the growth performance and intestinal health of subclinical necrotic enteritis (SNE)-challenged broilers. In total, 504 1-day-old Arbor Acres male chickens were selected and subsequently assigned into three treatments, including PC (basal diet + SNE challenge), PA (basal diet extra 10 mg/kg enramycin + SNE challenge), and PG (basal diet extra 3.20 × 10^9^ and 1.60 × 10^9^ CFU *B. licheniformis* per kg diet during 1–21 days and 22–42 days, respectively + SNE challenge). Results showed that *B. licheniformis* significantly decreased the intestinal lesion scores and down-regulated the *Claudin-3* mRNA levels in jejunum of SNE-infected broilers on day 25, but increased the *mucin-2* gene expression in broilers on day 42. In addition, *B. licheniformis* significantly up-regulated the mRNA levels of *TRIF* and *NF-κB* of SNE-challenged broilers compared with the control group on day 25 and *TLR-4*, *TRIF* compared with the control and the antibiotic group on day 42. The mRNA expression of growth factors (*GLP-2* and *TGF-β2*) and HSPs (*HSP60*, *HSP70*, and *HSP90*) were up-regulated in *B. licheniformis* supplementary group on days 25 and 42 compared with group PC. LEfSe analysis showed that the relative abundance of *Lachnospiraceae_UCG_010* was enriched in the PG group; nevertheless, *Clostridiales_vadinBB60* and *Rnminococcaceae_NK4A214* were in PA. PICRUSt analysis found that the metabolism of cofactors and vitamins, amino acid metabolism, and carbohydrate metabolism pathways were enriched, whereas energy metabolism, membrane transport, cell motility, and lipid metabolism were suppressed in *B. licheniformis*-supplemented groups as compared with the PC control. In conclusion, dietary supplementation of *B. licheniformis* alleviated the intestinal damage caused by SNE challenge that coincided with modulating intestinal microflora structure and barrier function as well as regulating intestinal mucosal immune responses.

## Introduction

Necrotic enteritis (NE) is an intestinal bacterial disease in poultry caused by *Clostridium perfringens* infection and annually costs up to six billion US dollars in production globally (Wade and Keyburn, 2015). *C. perfringens* is a spore-forming, strictly anaerobic Gram-positive bacterium which could produce up to 17 kinds of toxins (Parish, 1961). According to the secreted toxins, *C. perfringens* can be divided into five types: types A, B, C, D, and E. NE was caused by *C. perfringens* type A and/or C infection (Engström et al., 2003; Prescott et al., 2016). NE is typically divided into clinical NE and subclinical NE. Clinical NE usually exhibits mass death with a mortality rate up to 50% and causes intestinal ulcer erosion, bloody feces, and so on (Lee et al., 2011; Alnassan et al., 2014). However, SNE leads to mild intestinal damage in the flock, resulting in inappetence, malabsorption, poor digestion, and further impaired growth performance with a mortality generally less than 5% (Timbermont et al., 2011). Therefore, chronic intestinal mucosal damage in SNE-infected broilers causes more serious economic losses than clinical NE infections due to the difficulty in detection. Previous studies have demonstrated that NE infection is usually accompanied by intestinal lesions in broilers, disorders in intestinal microflora (Latorre et al., 2018), intestinal inflammation (Collier et al., 2008; Park et al., 2008), and damages of intestinal tight junction and mucus barrier in broilers (Golder et al., 2011; Forder et al., 2012; Guo et al., 2014). Therefore, modulation on intestinal health may be a great strategy to control NE infection in broiler.

In the post-antibiotic era, apart from plant extracts (Abudabos et al., 2017; Yin et al., 2017), organic acids (Song et al., 2017), polysaccharides (Tian et al., 2016), and vaccines (Mishra and Smyth, 2017), probiotics had been demonstrated to be an effective measure to promote animal growth (Khan and Naz, 2013; Mingmongkolchai and Panbangred, 2018). Evidences indicated that probiotics were one of the effective methods to prevent SNE infection in poultry for its protection on intestinal health (Venessa et al., 2016; Wang Y. et al., 2017). *Bacillus licheniformis* is a Gram-positive bacterium and characterized by high temperature and stress resistance. Previous studies had found that *B. licheniformis* could produce a variety of biologically active substances, such as digestive enzymes, lysozyme, bacteriocin, and antibacterial peptides, which promote animal performance by improving feed digestibility, stimulating the development of immune system, enhancing intestinal mucosal barrier function, inhibiting the colonization of pathogenic bacteria, promoting the proliferation of potentially beneficial microorganisms, and maintaining the balance of intestinal microflora (Rozs et al., 2001; Kim et al., 2004; Zhou et al., 2016). For example, Wang Y. et al. (2017) reported that *B. licheniformis* up-regulated the gene expression of tight junction proteins (TJP) and *mucin-2* in laying hens, thus maintaining the intestinal mechanical barrier and reducing intestinal permeability. Other research noted that probiotics *Bacillus* spp. strengthened host intestinal mucosal immunity through increasing the mRNA expression levels of TLRs, associated downstream adaptor proteins, and *NF-κB* in broiler chickens (Rajput et al., 2017), up-regulating the mRNA levels of cytokines and sIgA (Baikui et al., 2016). In addition, diets supplemented with *B. licheniformis* could also modulate the composition and structure of intestinal microbiota in broiler chickens challenged with NE (Lin et al., 2017; Xu et al., 2018). Some researchers have confirmed that probiotics *Bacillus* spp. as feed additives had achieved promising results in preventing and controlling NE infection in poultry (Jayaraman et al., 2013; Zhou et al., 2016; Wu et al., 2018). However, probiotic strains differ regarding their properties and clinical effects that they elicit; these differences are even observed when the strains belong to the same bacterial species. Therefore, the aim of this study was to explore whether *B. licheniformis* could alleviate the SNE infection similar to enramycin and reveal its action mechanism by determining intestinal barrier function, the immune responses as well as intestinal microflora.

## Materials and Methods

### Experimental Animals, Diets, and Treatments

A total of 504 1-day-old male Arbor Acres chicks with an average weight at 43.9 g (SD 0.87) were purchased from Beijing Arbor Acres Poultry Breeding Company (Beijing, China). On arrival, chicks were weighed and randomly assigned to three groups. Each group contained 12 replicates with 14 birds per replicate. Each replicate was reared in a separate isolator (240 × 60 × 60 cm^3^). The treatment groups were as follows: (1) positive control group (PC, basal diet + SNE infection); (2) antibiotic group (PA, basal diet extra 10 mg/kg enramycin + SNE infection); (3) *B. licheniformis*-treated group (PG, basal diet extra 3.20 × 10^9^ CFU *B. licheniformis* per kg diet [days 1–21], 1.60 × 10^9^ CFU *B. licheniformis* per kg diet [days 21–42] + SNE infection). *B. licheniformis* used in this study was provided by Chr. Hansen Co., Ltd. (Denmark) at a density of 3.20 × 10^9^ CFU/g. Antibiotic-free and coccidiostat-free corn–soybean meal basal diets were formulated according to National Research Council (1994) requirements for starter (days 1 to 21) and grower (days 22 to 42) periods. The composition and nutrient levels of the basal diet are presented in Table 1. The experimental diet was formulated by mixing the basal diet with *B. licheniformis* to reach 3.20 × 10^9^ and 1.60 × 10^9^ CFU/kg of diet in the starter and grower periods, respectively. To ensure the homogeneity of the additives, approximately 5 kg of the basal diet mixed with the additive was thoroughly mixed using a plastic bucket. Starter diets were pelleted and crumbled, whereas grower diets were just pelleted. All birds were reared in a farm and fed *ad libitum* and allowed to access water freely throughout the entire experimental period. Room temperature was maintained at 33°C during first 5 days and then gradually decreased by 2°C weekly until a final room temperature of 24°C was reached. Artificial light was provided in a 23 h light/1 h dark program. In addition, all birds were immunized with Newcastle disease virus vaccine plus infectious bronchitis virus vaccine via drinking water on day 21.

**TABLE 1:** Composition and nutrient levels of the basal diets.

**Items**	**Weeks 0–3**	**Weeks 4–6**
Ingredient, %		
Corn (CP 7.8%)	37.15	55.33
Wheat middlings	0	5.00
Wheat	20.00	0
Soybean meal (CP 46.8%)	34.00	31.00
Soybean oil	4.80	5.00
Limestone	0.90	0.70
Dicalcium phosphate	2.00	2.00
DL-Methionine, 98%	0.23	0.19
L-Lysine sulfate, 78%	0.15	0.10
Sodium chloride	0.30	0.30
Ethoxyquinoline, 33%	0.05	0.05
Choline chloride, 50%	0.24	0.15
Vitamin premix^*a*^	0.03	0.03
Mineral premix^*b*^	0.15	0.15
Total	100	100
Nutrient levels		
Metabolizable energy, Mcal/kg	3.03	3.10
Crude protein, %	21.77	19.80
Calcium, %	1.06	0.95
Non-phytate, %	0.45	0.42
Lysine, %	1.23	1.10
Methionine, %	0.52	0.46
Met + Cys, %	0.83	0.74

*^a^Vitamin premix provided per kilogram of complete diet: vitamin A 12,500 IU; vitamin D_3_ 2500 IU; vitamin E 30 IU; vitamin K_3_ 2.65 mg; vitamin B_12_ 0.025 mg; biotin 0.0325 mg; folic acid 1.25 mg; nicotinic acid 50 mg; pantothenic acid 12 mg; riboflavin 6 mg; thiamine mononitrate 2 mg.*

*^b^Mineral premix provided per kilogram of complete diet: iron 80 mg; copper 8 mg; manganese 100 mg; zinc 75 mg; iodine 0.35 mg; selenium 0.15 mg.*

### Experimental Induction of SNE

Avian *C. perfringens* type A strain CVCC2030 (China Veterinary Culture Collection Center, Beijing, China) was used for infection in this study. *C. perfringens* was anaerobically cultured in thioglycolate broth for 24 h at 37°C, then aseptically transferred into a cooked meat medium (CM605; Beijing Land Bridge Technology Co., Ltd.) supplemented with dried meat particles (CM607; Beijing Land Bridge Technology Co., Ltd.) and iron powders (Shanghai Kefeng Industry & Commerce Co., Ltd.) and incubated anaerobically for 18 h at 37°C. Establishment of SNE model in broilers referenced Wu et al. (2018) with little modifications. Briefly, each bird in this study was orally gavaged with 12,000 *Eimeria maxima* oocysts (College of Veterinary Medicine, China Agricultural University, Beijing, China) at 12 days of age, and subsequently with 1 ml of *C. perfringens* (1 × 10^9^ CFU/ml) once a day during days 17 to 23 to establish the SNE model.

### Growth Performance

On days 21 and 42, the body weight (BW) and feed intake of each replicate were recorded. Then the average gain (AG), feed intake (FI), and feed conversion ratio (FCR) were calculated for days 1–21, 22–42, and 1–42, respectively. Death of birds in each replicate was recorded daily and was used for determining the mortality.

### Intestinal Lesion Score and Sample Collection

On days 25 and 42, one bird per replicate was randomly selected, weighed, and euthanized by jugular exsanguination. The middle segments of jejunum (approximately 1 cm) were cut off carefully and gently rinsed with ice-cold sterile saline to remove internal digesta. Subsequently, the jejunum segments were fixed in 4% paraformaldehyde immediately for further morphology analysis. Another jejunum sample was collected and washed, then frozen in liquid nitrogen immediately and stored at −80°C for the subsequent gene expression analysis. Liver and cecal digesta samples were put into sterile tubes, snap-frozen in liquid nitrogen, and transferred to −80°C. Liver samples were used to determine microbial translocation, while cecal samples were used to determine bacterial populations, short-chain fatty acid (SCFA) contents, and DNA extraction. The duodenum, jejunum, and ileum of each bird were cut longitudinally and scored 0 (none) to 4 (severe) for NE gut lesions by three independent observers blindly as previously described by Gholamiandehkordi et al. (2007) with some modifications.

The scoring criteria are as follows: 0 = no obvious lesions; 1 = thin and friable intestine with hemorrhagic spots (1–5 foci); 2 = small gas production and focal necrosis or ulceration (hemorrhagic spots 6–15); 3 = gas-filled intestine and patches of necrosis 1 to 2 cm long; 4 = diffuse necrosis with great amounts of gas in the intestine.

### Bacterial Population of Cecal Digesta and Liver Bacterial Translocation

Quantification of bacterial population in cecal digesta (or liver) was done with techniques as previously described (Wu et al., 2018). Briefly, approximately 1 g of each sample was diluted with 9 ml ice-cold sterile buffered peptone water (CM201; Land Bridge Technology Ltd.) and homogenized. The homogenized suspension of each sample was serially diluted up to 10^–7^, then 100 μl of each dilution was plated on selective ager plates for bacterial quantification. Each sample was plated in duplicate. Commercial media were used for cultivation of *C. perfringens* (tryptose–sulfite–cycloserine agar, TSC, CM 138; Beijing Land Bridge Technology Co., Ltd.), coliform bacteria (Eosin–Methylene Blue Agar, EMB, CM105; Beijing Land Bridge Technology Co., Ltd.) and lactic acid bacteria (de Man, Rogosa, and Sharpe agar, MRS, CM 188; Land Bridge Technology Co., Ltd.). *C. perfringens* and lactic acid bacteria were incubated anaerobically for 48 h at 37°C, while coliform bacteria were incubated aerobically for 24 h at 37°C. The number of colony-forming units was expressed as a logarithmic transformation per gram of cecal digesta (or liver).

### Intestinal Morphology Observation and Analysis

Fixed jejunum tissues were embedded in paraffin, then sliced into 5-μm thickness, deparaffinized in xylene, rehydrated, and mounted on glass slides. Periodic acid–Schiff (PAS) stain was used to stain the sections for determining the number of goblet cells, whereas H&E stain was used for villous morphology measurement. Five intact villi in every slide were chosen for measurement of goblet cells, villus height (VH), and crypt depth (CD) with Image-pro plus 6.0 (Media Cybernetics, Inc., Rockville, MD, United States) at ×40 magnification. The means of villus height and crypt depth were calculated and subsequently were used to obtain the VH/CD.

### Gene Expression in Jejunum

Extraction of total RNA in jejunum was performed using Trizol reagent (Invitrogen Life Technologies, Carlsbad, CA, United States) according to the manufacturer’s instructions. The concentration and purity of total RNA were determined by using a NanoDrop-2000 spectrophotometer (Thermo Fisher Scientific, Waltham, MA, United States). Then, complementary DNA (cDNA) was synthesized by using Primer Script RT Reagent kit (Takara Bio Inc.) according to the manufacturer’s instructions. Using the synthesized cDNA as a template, quantitative real-time PCR (qRT-PCR) was performed in Applied Biosystems’ 7500 Fast Real-Time PCR System with SYBR Premix Ex Taq kit (Takara Bio Inc.) in accordance with the manufacturer’s guidelines. Thermocycling protocol was as follows: 95°C for 30 s, followed by 40 cycles of 95°C for 5 s and 60°C for 34 s for denaturation and annealing/extension, respectively. The purity and specificity of PCR products were determined by melt curve analysis. All data were analyzed using the 2^–ΔΔ*Ct*^ method, and glyceraldehyde 3-phosphate dehydrogenase (*GAPDH*) and *β-actin* were used to normalize the relative mRNA levels (Livak and Schmittgen, 2001). All samples (*n* = 6) from each group on days 25 and 42 were done in triplicate. Target genes include TJP genes (*Occludin*, *Claudin-1*, *Claudin-3*, Zonula occludens-1[*ZO-1*], *mucin-2*), TLR signal pathway-related genes (*TLR-4*, *TLR-2*, *TRIF*, *MyD88*, *NF-κB*, *IL-1β*, *IL-10*, *IL-17*, *IFN-γ*, *TNF-α*), heat shock protein genes (*HSP60*, *HSP70*, *HSP90*), and growth factor genes (*IGF-2*, *GLP-2*, *TGF-β2*). Primers of target genes used in this study are presented in Supplementary Tables 1, 2.

### SCFA Concentration in Cecal Content

A total of 0.5–1.0 g cecal digesta from day 42 sample was weighed and put into a 10-ml polypropylene tube with 8 ml deionized water, then an ultrasonic bath was performed for 30 min, the suspension subsequently was centrifuged at 8000 rpm for 10 min. The supernatant was collected and diluted 10-fold, and then filtered with a 0.22-μm filtrator. Next, 25 μl of filtered solution was subjected to high-performance ion chromatography system (ICS-3000; Dionex, United States) for conductivity detection analysis. Organic acids were separated on an AS11 analytical column (250 × 4 mm^2^) and an AG11 guard column under the following gradient conditions (the gradient was based on potassium hydroxide): 0–5 min, 0.8–1.5 mM; 5–10 min, 1.5–2.5 mM; and 10–15 min, 2.5 mM; the flow rate was 1.0 ml/min. The results of SCFAs were expressed as milligrams per kilogram of digesta.

### Microbial DNA Extraction, 16S rRNA Gene Amplification, Sequencing, and Bioinformatics Analysis

Bacterial DNA extraction of day 25 cecal digesta was performed by using PowerSoil DNA Isolation Kit (ANBIOSCI Tech Ltd., United States) according to the manufacturer’s instructions. Integrity of DNA was appraised by agarose gel electrophoresis, then the qualified DNA was used as template for the V3–V4 region of bacterial 16S rRNA gene amplification with barcoded primer pair 338F: 5′-ACTCCTACGGGAGGCAGCA-3′ and 806R: 5′-GGACTACHVGGGTWTCTAAT-3′. The KAPA HiFi Hotstart ReadyMix PCR kit (Kapa Biosystems, United States) was used in the PCR amplification and the procedures were as follows: 98°C for 2 min (1 cycle), 98°C for 30 s/50°C for 30 s/72°C for 1 min (25 cycles), and finally 72°C lasts for 5 min. The amplification products were determined by 2% agarose gel and purified with AxyPrep DNA Gel Extraction Kit (Axygen Biosciences, Union City, CA, United States). Amplicon libraries were sequenced on Illumina HiSeq 2500 platform (Illumina, San Diego, CA, United States) at Biomarker Technologies Co., Ltd. (Beijing, China). The sequencing data were merged using FLASH (version 1.2.11) to get raw tags. Raw tags were then subjected to filtration (Trimmomatic, version 0.33) and chimera sequences removed (UCHIME, version 8.1) to obtain effective tags. UCLUST (Edgar, 2010) was used to cluster effective tags into operational taxonomic units (OTUs) at a similarity level of 97% with QIIME software (version 1.8.0) (Caporaso et al., 2010). Afterward, basing on the Silva taxonomic database, OTUs were annotated. Venn diagram, rarefaction curve, and bacteria relative abundance were created with R software (version 2.15.3). Alpha diversity, including ACE, Chao1, Simpson, and Shannon index, were investigated by Mothur (version 1.30), and the significance of these items was determined using a Mann–Whitney *U* test. β Diversity was calculated from binary_jaccard distance (PERMANOVA/ANOSIM analysis) in QIIME software. A two-sided Student’s *t*-test was used to determine the significance of the differences between groups. Line discriminant analysis (LDA) effect size (LEfSe^1^) (Segata et al., 2011) tool was used to determine statistically different biomarkers between groups (LDA value: 2) based on the taxonomic files obtained from the QIIME analysis. The raw sequences used in our study had been uploaded at the Sequence Read Archive of the National Center for Biotechnology Information, with the study accession number PRJNA574872. The functions of the cecum metagenomes were predicted using PICRUSt (Phylogenetic investigation of communities by reconstruction of unobserved states) analysis based on high-quality sequences (Langille et al., 2013).

### Statistical Analysis

All results were displayed as means ± SEM. Statistical significance of growth performance, intestinal lesion scores, bacterial population, intestinal morphology, gene expression, and SCFA content were determined by one-way ANOVA, followed by Duncan’s multiple comparison test (SPSS, version 20.0, Chicago, IL, United States). Kruskal–Wallis test was employed to analyze the difference in bacterial relative abundance. Significant difference was declared when *P* < 0.05.

## Results

### Growth Performance

We measured four indexes concerning broiler chickens’ productivity as shown in Table 2. There was no significant difference in BW, AG, FI, and FCR between groups, while numerically higher BW, AG, and lower FCR were observed in PA and PG groups when compared with PC group at days 21–42 and days 1–42, and the value of those indexes of PA and PG group were close to each other. There was no significant difference in mortality among groups.

**TABLE 2:** Effects of *B. licheniformis* and enramycin on growth performance of broilers challenged with SNE.

**Items**	**PC**	**PA**	**PG**	**SEM**	***P* values**
Days 1–21					
BW (g/bird)	641	652	640	5.7	0.623
AG (g/bird)	598	609	597	5.7	0.624
FI (g/bird)	856	866	853	6.4	0.694
FCR	1.43	1.42	1.43	0.005	0.631
Mortality (%)	0.00	1.19	0.60	0.439	0.555
Days 21–42					
BW (g/bird)	2346	2394	2395	11.1	0.103
AG (g/bird)	1705	1734	1753	10.8	0.178
FI (g/bird)	2996	2991	3036	14.4	0.384
FCR	1.76	1.73	1.73	0.008	0.221
Mortality (%)	1.19	1.19	0.60	0.418	0.807
Days 1–42					
BW (g/bird)	2346	2394	2395	11.1	0.103
AG (g/bird)	2303	2351	2352	11.1	0.103
FI (g/bird)	3852	3865	3892	16.8	0.614
FCR	1.67	1.64	1.65	0.006	0.101
Mortality (%)	1.19	2.38	1.19	0.577	0.636

*Data are presented as means ± SEM (*n* = 12; cage was used as experimental unit). PC, basal diet + SNE; PA, basal diet extra antibiotics + SNE; PG, basal diet extra *B. licheniformis* + SNE.*

*BW, body weight; AG, average gain; FI, feed intake; FCR, feed conversion rate (FI/AG).*

### Small Intestine Lesion Scores and Jejunum Morphology

As shown in Table 3, lesion scores of duodenum and small intestine in PG group were significantly lower than those in PC and PA group and jejunum lesion score in PG group was lower than PA group on 2 days post-infection (DPI) (*P* < 0.05). Similarly, there was a tendency that lesion scores of ileum in PG were lower than in PC and PA groups (0.05 < *P* < 0.1). In addition, lesion scores of duodenum, jejunum, and small intestine in PA group were comparable with PC group. At 42 days of age (19 DPI), no significant difference was detected of intestinal lesion scores in the three experiment groups. We also investigated the jejunum morphology of broilers at 2 DPI (Table 4). It was found that the number of goblet cells were significantly decreased in PA group compared with PC and PG group (*P* < 0.05). However, the jejunum villus height (VH), crypt depth (CD), and VH/CD values were not changed significantly among groups.

**TABLE 3:** Effects of *B. licheniformis* and enramycin on intestinal lesion scores of broilers challenged with SNE.

**Items**	**PC**	**PA**	**PG**	**SEM**	***P* values**
Day 25					
Duodenum	1.13^a^	1.08^a^	0.38^b^	0.081	0.000
Jejunum	0.75^ab^	1.04^a^	0.67^b^	0.060	0.022
Ileum	0.29	0.38	0.08	0.061	0.097
Small intestine	2.17^a^	2.50^a^	1.13^b^	0.927	0.000
Day 42					
Duodenum	0.58	0.42	0.50	0.049	0.378
Jejunum	0.29	0.29	0.25	0.051	0.856
Ileum	0.04	0.00	0.13	0.027	0.147
Small intestine	0.92	0.71	0.88	0.080	0.541

*Data are presented as means ± SEM (*n* = 12; cage was used as experimental unit). PC, basal diet + SNE; PA, basal diet extra antibiotics + SNE; PG, basal diet extra *B. licheniformis* + SNE. Means within a row lacking a common superscript differ significantly (*P* < 0.05).*

**TABLE 4:** Effects of *B. licheniformis* and enramycin on jejunal morphology of 25-day-old broilers challenged with SNE.

**Items**	**PC**	**PA**	**PG**	**SEM**	***P* values**
Goblet cells	194.8^a^	149.5^b^	204.6^a^	8.196	0.005
VH (μm)	706.8	692.3	738.1	30.014	0.828
CD (μm)	180.5	191.8	196.4	6.964	0.953
VH/CD	4.12	3.65	3.68	0.159	0.459

*Data are presented as means ± SEM (*n* = 6; cage was used as experimental unit). PC, basal diet + SNE; PA, basal diet extra antibiotics + SNE; PG, basal diet extra *B. licheniformis* + SNE. Means within a row lacking a common superscript differ significantly (*P* < 0.05).*

*Goblet cells, the number of goblet cells on a villus; VH, villus height; CD, crypt depth.*

### Cecal Bacterial Population and Liver *Clostridium perfringens* Translocation

Table 5 shows the results of cecal bacterial population and liver *C. perfringens* translocation. At 25 days of age (2 DPI), SNE-infected birds fed diets supplemented with enramycin exhibited significantly reduced population of *C. perfringens* in cecum and liver in contrast to that in PC and PG groups (*P* < 0.05). However, probiotic supplementation failed to decrease the population of *C. perfringens* in both cecum and liver. Furthermore, no significant differences were observed in *Escherichia coli* and *Lactobacilli* population in cecum between PC, PA, and PG groups. At 42 days of age, the cecal *Escherichia coli* population in the PA group was significantly higher than that in the PC and PG groups (*P* < 0.05). Similarly, the difference between PG and PC group was not statistically significant.

**TABLE 5:** Effects of *B. licheniformis* and enramycin the amounts of bacteria (lg CFU/g^1^) in cecal digesta of broilers challenged with SNE.

	**Items**	**PC**	**PA**	**PG**	**SEM**	***P* values**
Day 25						
Cecal digesta	*Clostridium perfringens*	4.62^a^	1.75^b^	4.11^a^	0.423	0.004
	*Escherichia coli*	6.67	7.51	6.52	0.229	0.166
	*Lactobacillus*	10.15	9.83	9.48	0.222	0.495
Liver	*Clostridium perfringens*	1.56^a^	0.17^b^	1.46^a^	0.233	0.011
Day 42						
Cecal digesta	*Clostridium perfringens*	1.24	1.53	0.90	0.270	0.657
	*Escherichia coli*	6.75^b^	7.73^a^	6.83^b^	0.154	0.007
	*Lactobacillus*	8.69	8.16	8.26	0.178	0.469
Liver	*Clostridium perfringens*	0.38	0.17	0.27	0.127	0.804

*Data are presented as means ± SEM (*n* = 6; cage was used as experimental unit). PC, basal diet + SNE; PA, basal diet extra antibiotics + SNE; PG, basal diet extra *B. licheniformis* + SNE. Means within a row lacking a common superscript differ significantly (*P* < 0.05).*

*^1^lg CFU/g, log 10 colony-forming units per gram of cecal digesta.*

### Jejunal Tight Junction Protein Gene and Mucin Gene Expression

As depicted in Table 6, the expression of *Claudin-3* in the jejunum of SNE-infected chickens fed with *B. licheniformis* was lower than that in the PC group at day 25 (*P* < 0.05), but no significant difference was seen between PG and PA group. Only a downtrend of *Claudin-3* levels was detected in PA group when compared with PC group. At 42 days of age, *Claudin-3* mRNA levels in PA group were significantly up-regulated when compared with the PC and PG groups (*P* < 0.05). Besides, a tendency was detected that the *ZO-1* expression of PA and PG group was higher than PC group (0.05 < *P* < 0.1). Similar with the results of the number of goblet cells on villus, *mucin-2* expression levels in the PA and PG groups were significantly increased when compared with the PC group at day 42 (*P* < 0.05). Moreover, the gene expression of *mucin-2* in the PG group was much higher than that in the antibiotic supplemented group (*P* < 0.05).

**TABLE 6:** Effects of *B. licheniformis* and enramycin on tight junction protein and *mucin-2* gene expression in jejunum of broilers challenged with SNE.

**Items**	**PC**	**PA**	**PG**	**SEM**	***P* values**
Day 25					
*Occludin*	1.03	0.82	0.83	0.048	0.128
*Claudin-1*	1.08	1.05	0.87	0.081	0.555
*Claudin-3*	1.01^a^	0.87^b^	0.72^b^	0.045	0.026
*ZO-1*	1.02	1.18	0.93	0.059	0.239
*Mucin-2*	1.01	0.94	1.01	0.036	0.677
Day 42					
*Occludin*	1.02	1.13	1.12	0.049	0.629
*Claudin-1*	1.03	1.04	0.90	0.051	0.505
*Claudin-3*	1.01^b^	1.48^a^	1.10^b^	0.073	0.008
*ZO-1*	1.01	1.22	1.23	0.044	0.064
*Mucin-2*	1.04^c^	1.79^b^	2.80^a^	0.202	0.000

*Data are presented as means ± SEM (*n* = 6; cage was used as experimental unit). PC, basal diet + SNE; PA, basal diet extra antibiotics + SNE; PG, basal diet extra *B. licheniformis* + SNE. Means within a row lacking a common superscript differ significantly (*P* < 0.05).*

*ZO, zonula occludens.*

### Jejunal Toll-Like Receptors Signaling Pathway and Immune-Related Cytokine Gene Expression

Table 7 presents the results of jejunal TLRs signaling pathway and immune-related cytokine gene expression in broilers. On day 25, dietary antibiotics and *B. licheniformis* significantly up-regulated the mRNA levels of *TRIF* and *NF-κB* in jejunum of SNE-infected broiler chickens compared with the PC group (*P* < 0.05). Moreover, the mRNA expression level of *IL-17* gene was significantly up-regulated in the PA group than that in the PC and PG groups (*P* < 0.05). On day 42, jejunal mRNA expression levels of *TLR-4*, *TRIF*, and *NF-κB* were significantly increased in the PG group when compared with the PC and PA groups (*P* < 0.05). Interestingly, birds fed diet supplemented with antibiotics had significantly lower *IL-17* and *TRIF* mRNA levels in contrast to the PC group (*P* < 0.05). In addition, supplementation of *B. licheniformis* also significantly up-regulated *IL-1β* mRNA levels compared with the PA group (*P* < 0.05).

**TABLE 7:** Effects of *B. licheniformis* and enramycin on TLR signal pathway-related gene expression in jejunum of broilers challenged with SNE.

**Items**	**PC**	**PA**	**PG**	**SEM**	***P* values**
Day 25					
*TLR-4*	1.02	1.35	1.29	0.077	0.174
*TLR-2*	1.02	1.21	1.18	0.077	0.592
*TRIF*	1.00^b^	1.24^a^	1.33^a^	0.045	0.002
*MyD88*	1.07	1.17	0.88	0.067	0.227
*NF*-κ*B*	1.01^b^	1.52^a^	1.42^a^	0.080	0.012
*IL*-1β	1.11	1.85	1.66	0.218	0.378
*IL-10*	1.07	1.19	0.90	0.094	0.475
*IL-17*	1.10^b^	2.72^a^	1.40^b^	0.262	0.021
*IFN*-γ	1.05	1.12	1.05	0.089	0.932
*TNF*-α	1.02	1.37	1.35	0.074	0.084
Day 42					
*TLR-4*	1.02^b^	1.04^b^	1.52^a^	0.075	0.002
*TLR-2*	1.05	0.84	1.10	0.068	0.299
*TRIF*	1.02^b^	0.76^c^	1.30^a^	0.061	0.000
*MyD88*	1.01	1.09	0.98	0.045	0.590
*NF*-κ*B*	1.04^b^	1.16^b^	1.48^a^	0.072	0.024
*IL*-*1*β	1.03^ab^	0.61^b^	1.17^a^	0.097	0.039
*IL-10*	1.07	0.74	1.04	0.092	0.276
*IL-17*	0.98^a^	0.45^b^	0.99^a^	0.097	0.016
*IFN*-γ	1.03	0.85	0.96	0.055	0.445
*TNF*-α	1.04	1.13	1.35	0.066	0.151

*Data are presented as means ± SEM (*n* = 6; cage was used as experimental unit). PC, basal diet + SNE; PA, basal diet extra antibiotics + SNE; PG, basal diet extra *B. licheniformis* + SNE. Means within a row lacking a common superscript differ significantly (*P* < 0.05).*

### Gene Expression of Jejunal Heat Shock Proteins and Growth Factors

On day 25, when compared with the PC group, addition of antibiotics in feed significantly up-regulated the relative gene expressions of *HSP90*, *IGF-2*, *GLP-2*, and *TGF-β2* in the jejunum of SNE-infected broilers (*P* < 0.05, Table 8). Meanwhile, higher mRNA levels of *HSP60*, *HSP90*, and *GLP-2* were also detected in the PG group when compared with the PC group (*P* < 0.05). On day 42, dietary antibiotics had significantly lower mRNA levels of *HSP90* than that in PC group (*P* < 0.05), whereas birds fed diets supplemented with *B. licheniformis* exhibited significantly higher gene expression of *HSP60*, *HSP70*, *HSP90*, *GLP-2*, and *TGF-β2* when compared with the PC and PA groups (*P* < 0.05).

**TABLE 8:** Effects of *B. licheniformis* and enramycin on recovery protein gene expression in jejunum of broilers challenged with SNE.

**Items**	**PC**	**PA**	**PG**	**SEM**	***P* values**
Day 25					
*HSP60*	1.04^b^	1.24^b^	2.53^a^	0.183	0.000
*HSP70*	1.03	1.17	1.19	0.046	0.309
*HSP90*	1.01^c^	1.83^b^	2.51^a^	0.177	0.000
*IGF-2*	1.04^b^	1.65^a^	1.05^b^	0.102	0.010
*GLP-2*	1.04^b^	1.81^a^	2.22^a^	0.175	0.010
*TGF*-β*2*	1.02^b^	1.65^a^	1.37^ab^	0.097	0.018
Day 42					
*HSP60*	1.03^b^	1.13^b^	1.58^a^	0.075	0.001
*HSP70*	1.01^b^	0.95^b^	1.37^a^	0.055	0.000
*HSP90*	1.02^b^	0.65^c^	1.37	0.083	0.000
*IGF-2*	1.04	1.13	1.47	0.093	0.141
*GLP-2*	1.09^*b*^	1.34^b^	1.83^a^	0.108	0.011
*TGF*-β*2*	1.02^b^	1.24^b^	1.65^a^	0.081	0.001

*Data are presented as means ± SEM (*n* = 6; cage was used as experimental unit). PC, basal diet + SNE; PA, basal diet extra antibiotics + SNE; PG, basal diet extra *B. licheniformis* + SNE. Means within a row lacking a common superscript differ significantly (*P* < 0.05).*

*HSP, heat shock protein; IGF, insulin-like growth factors; GLP, glucagon-like peptide.*

### Short-Chain Fatty Acids in Cecal Content

On day 42, we investigated the SCFA concentrations in cecal content of SNE-infected broilers (Table 9). Significant changes were only observed in the formic acid concentration between groups. The concentration of formic acid in the PA and PG groups were significantly increased compared with the PC group (*P* < 0.05), but no significant differences were detected between PA and PG groups (*P* > 0.05).

**TABLE 9:** Effects of *B. licheniformis* and enramycin on concentration of short-chain fatty acids in cecal content of 42-day-old broilers (mg/kg).

**Items**	**PC**	**PA**	**PG**	**SEM**	***P* values**
Lactic acid	297.2	216.6	390.1	69.34	0.619
Formic acid	215.7^b^	276.1^a^	273.9^a^	12.74	0.027
Acetic acid	3958.7	3886.8	3737.9	138.68	0.822
Propionic acid	2467.9	1843.1	2091.2	135.81	0.170
Butyric acid	1584.2	1284.4	1415.1	97.46	0.480
Isobutyric acid	61.5	52.9	69.3	6.780	0.655
Valeric acid	121.7	101.7	125.1	6.547	0.305
Isovaleric acid	66.0	92.7	73.2	9.328	0.508

*Data are presented as means ± SEM (*n* = 6; cage was used as experimental unit). PC, basal diet + SNE; PA, basal diet extra antibiotics + SNE; PG, basal diet extra *B. licheniformis* + SNE. Means within a row lacking a common superscript differ significantly (*P* < 0.05).*

### Cecal Microbiome

A total of 1,440,717 pairs of reads were generated after 16S rRNA sequencing of 18 cecal digesta samples. Then, we obtained 1,191,203 effective Tags after splicing, filtering, and removal of chimeras, and an average of 66,178 effective Tags were obtained from each sample. Based on 97% sequence similarity, Tags were clustered into 510 OTUs, of which 475 OTUs were shared by three groups, and only 2, 4, and 1 OTUs were exclusive in PC, PA, and PG groups, respectively (Supplementary Figure 1). Furthermore, the alpha diversity analysis of cecal microbiota showed that no significant difference in ACE, Chao1, Simpson, and Shannon index between groups (*P* > 0.05 and Table 10).

**TABLE 10:** Effects of *B. licheniformis* and enramycin on alpha diversity of cecal microbiota of 25-day-old broilers challenged with SNE.

**Items**	**PC**	**PA**	**PG**	**SEM**	***P* values**
ACE	417.1	365.8	393.1	15.123	0.407
Chao1	423.6	384.7	397.5	19.036	0.280
Simpson	0.115	0.126	0.101	0.019	0.870
Shannon	3.46	3.21	3.40	0.167	0.746

*Data are presented as means ± SEM (*n* = 6; cage was used as experimental unit). PC, basal diet + SNE; PA, basal diet extra antibiotics + SNE; PG, basal diet extra *B. licheniformis* + SNE. Means within a row lacking a common superscript differ significantly (*P* < 0.05).*

**FIGURE 1:**
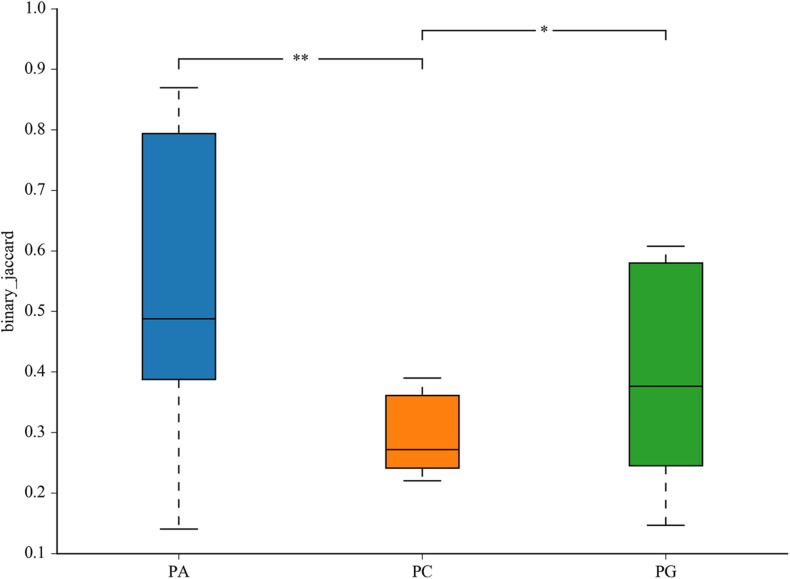
Differential cecum microbiota community (β diversity) between groups in 25-day-old broilers. PC, basal diet + SNE; PA, basal diet extra antibiotics + SNE; PG, basal diet extra *B. licheniformis* + SNE. Values are means with their standard errors. *P < 0.05, **P < 0.01.

The representative sequences of OTUs were annotated with Silva database. Then we analyzed the bacterial composition in phylum and genus level of samples. The most abundant (top 6) phyla of bacteria are presented in Supplementary Figure 2A. At the phylum level, the cecal microbiota was dominated by *Firmicutes*, *Bacteroidetes*, *Proteobacteria*, and *Tenericutes*, together accounting for over 99.7% of the total sequences. However, no significant differences were detected in those phyla between groups (*P* > 0.05, Table 11). The top 10 abundant bacteria in genus level were *Faecalibacterium*, *Lactobacillus*, *Barnesiella*, *[Ruminococcus]_torques_group*, *Alistipes*, *Ruminococcaceae_UCG-014*, *uncultured_bacte rium_f_Lachnospiraceae*, *uncultured_bacterium_f_Rumi-noc occaceae*, *Bacteroides*, and *Megamonas*. The relative abundance of Others and Unclassified bacterium were 36.0% (Supplementary Figure 2B). Similarly, no significant differences were detected in abundance of those genera between groups (*P* > 0.05, Table 12). Then binary_jaccard algorithm for PERMANOVA/ANOSIM analysis (Beta diversity box plot, Figure 1) was used to evaluate differences in cecal bacterial community structure between different groups. As shown in Figure 1, β diversity of the PC group was significantly different from the PA and PG groups; nevertheless, no significant differences were detected in β diversity between the PA and PG groups.

**TABLE 11:** Effects of *B. licheniformis* and enramycin on relative abundances of phyla in cecal microbiota of 25-day-old broilers challenged with SNE (%).

**Items**	**PC**	**PA**	**PG**	**SEM**	***P* values**
*Firmicutes*	72.68	72.41	86.55	3.751	0.203
*Bacteroidetes*	23.27	20.71	10.47	3.938	0.519
*Proteobacteria*	2.20	4.61	1.01	1.012	0.325
*Tenericutes*	1.68	1.84	1.70	0.388	0.805
*Actinobacteria*	0.12	0.44	0.26	0.094	0.805

*Data are presented as means ± SEM (*n* = 6; cage was used as experimental unit). PC, basal diet + SNE; PA, basal diet extra antibiotics + SNE; PG, basal diet extra *B. licheniformis* + SNE.*

**TABLE 12:** Effects of *B. licheniformis* and enramycin on relative abundances of genus in cecal microbiota of 25-day-old broilers challenged with SNE (%).

**Items**	**PC**	**PA**	**PG**	**SEM**	***P* values**
*Faecalibacterium*	11.29	10.89	17.44	2.955	0.366
*Lactobacillus*	6.38	15.43	5.75	4.093	0.414
*Barnesiella*	12.68	9.45	0.17	3.304	0.116
*[Ruminococcus]_torques_group*	5.67	5.34	8.33	1.024	0.587
*Alistipes*	5.21	3.58	8.60	1.462	0.399
*Ruminococcaceae_UCG-014*	5.57	4.27	6.67	0.817	0.484
*uncultured_bacterium_f_Lachnospiraceae*	3.95	4.60	5.13	0.762	0.738
*uncultured_bacterium_f_Ruminococcaceae*	5.58	4.10	3.71	0.601	0.444
*Bacteroides*	4.26	7.06	0.01	1.364	0.244
*Megamonas*	0.00	2.47	8.37	1.954	0.419

*Data are presented as means ± SEM (*n* = 6; cage was used as experimental unit). PC, basal diet + SNE; PA, basal diet extra antibiotics + SNE; PG, basal diet extra *B. licheniformis* + SNE. Means within a row lacking a common superscript differ significantly (*P* < 0.05).*

**FIGURE 2:**
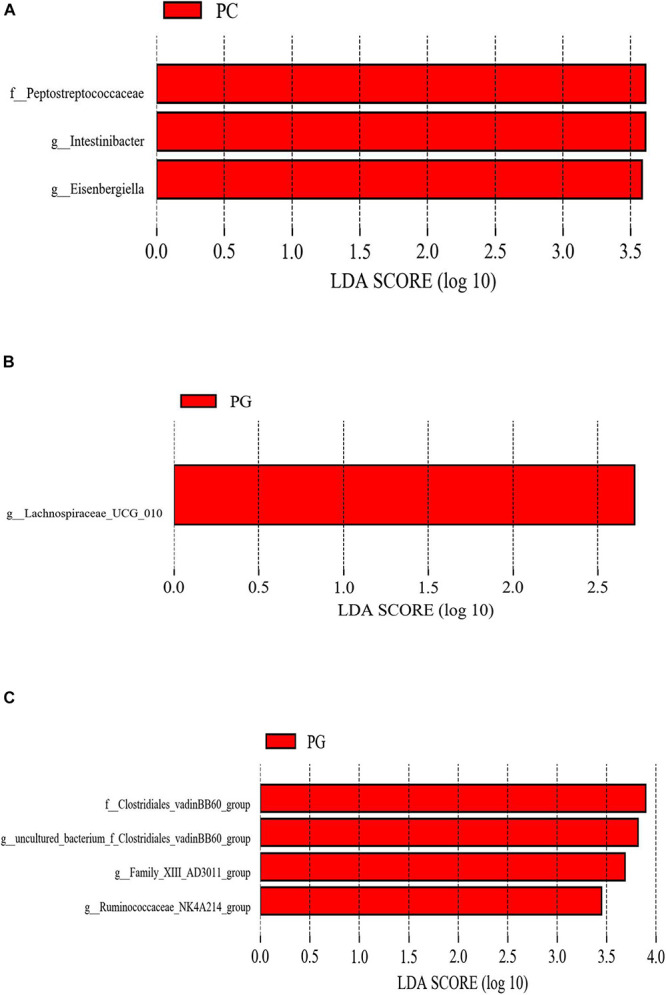
The different phylotypes differed between groups using LEfSe analysis. These figures show the bacteria of which the LDA Score is greater than the set value (the default setting is 2.0) between groups PC and PA. **(A)** Groups PC and PG. **(B)** Groups PG and PA. **(C)** The length of the histogram represents the size of the difference species (i.e., LDA Score), and the different colors represent the different groups. PC, basal diet + SNE; PA, basal diet extra antibiotics + SNE; PG, basal diet extra *B. licheniformis* + SNE.

LEfSe analysis was used to determine the statistically different biomarkers between groups. As presented in Figure 2, when compared with the PC group, *Peptostreptococcaceae*, *Intestinibacter*, and *Eisenbergiella* were less abundant in the PA group (Figure 2A); nevertheless, *Lachnospiraceae_UCG_010* were enriched in the PG group when compared with group PC (Figure 2B). Furthermore, *Clostridiales_vadinBB60_group*, *g_uncultured_bacterium_f_Clostridiales_vadinBB60_group*, *Family_XIII_AD3011_group*, and *Ruminococcaceae_NK4A214_group* were more abundant in the PG group in contrast to the PA group (Figure 2C).

### Predicting the Function of Intestinal Bacteria

**FIGURE 3:**
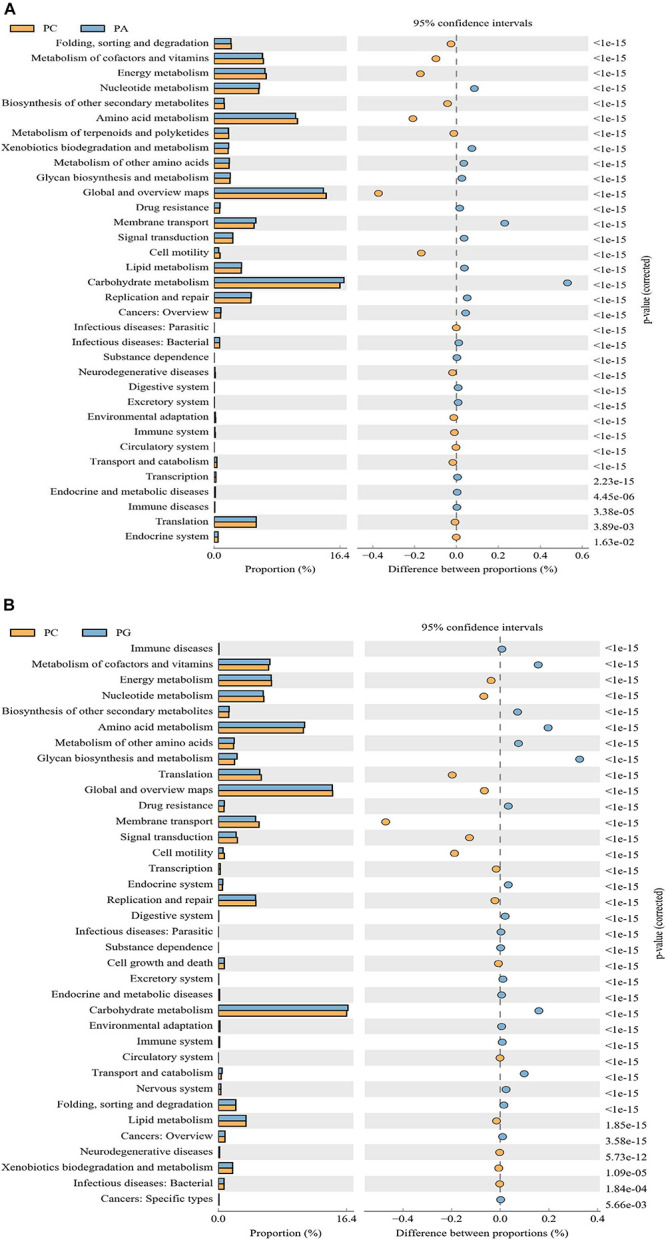
The microbial pathways grouped into level-2 functional categories using PICRUSt. PC between groups PC and PA **(A)**, and groups PC and PG **(B)**. PC, basal diet + SNE; PA, basal diet extra antibiotics + SNE; PG, basal diet extra *B. licheniformis* + SNE.

PICRUSt analysis showed a significant functional gene difference between group PC and PA or PG (Figure 3). We found that six pathways were enriched in group PC and eight pathways were in group PA and PG altogether. Besides, 10 pathways were enriched in group PA and 12 pathways in group PA uniquely explaining the fact that the functional profiles representing the microbial communities in group PA and PG were relatively similar and different. Notably, metabolic pathways were mostly common among the significantly differentially represented pathways, which indicated the different metabolic status between groups. Comparing with the group PA, energy metabolism, amino metabolism, and cell motility were enriched in the PC group (*P* < 0.01, Figure 3A), whereas carbohydrate metabolism, nucleotide metabolism, xenobiotics biodegradation and metabolism, and membrane transport were significantly enriched in PA group (*P* < 0.001). Compared with the group PG, translation, membrane transport, signal transduction, and cell motility were enriched in the PC group (*P* < 0.001, Figure 3B), and metabolism of cofactors and vitamins, biosynthesis of other secondary metabolites, amino acid metabolism, folding sorting and degradation, endocrine system, excretory system, immune system, nervous system, transport, and catabolism were enhanced in group PG (*P* < 0.001).

## Discussion

Necrotic enteritis caused by *C. perfringens* infection destroys the gut integrity of chickens and seriously damages the intestinal function of poultry, leading to a decline in the growth performance (Abudabos et al., 2017). Related studies had shown that probiotics play a positive role on animal health and prevention of NE diseases. Zhou et al. (2016) reported that the body weight decreased significantly after broilers were infected with NE, while FCR increased. However, the improved growth performance was observed when broilers were pre-treated with *B. licheniformis* H2. Whelan et al. (2018) also demonstrated that *Bacillus subtilis* DSM32315 alleviated the adverse impact on growth performance of broiler chickens caused by NE infection. Interestingly, Lin et al. (2017) found that diets supplemented with *B. licheniformis* H2 which was used in Zhou et al. (2016) research had no significant improvement on body weight, feed intake, and FCR of *C. perfringens* challenged broilers. In accordance with Lin’s study, our results showed that pre-treatment with *B. licheniformis* had no improvement on growth performance of SNE-infected broilers. *B. licheniformis* was believed to prevent NE in broilers, but the mechanism of action of *B. licheniformis* as a probiotic for the control and prevention of NE is not totally clear. In addition, the function of *B. licheniformis* may differ from different strains and dietary concentrations (Knap et al., 2010). Thus, the optimum application rate of *B. licheniformis* used in studies may need further identified. Furthermore, differences in housing environment, broiler breed, administration route of probiotic *B. licheniformis* as well as the way to establish NE model may influence the outcome of *B. licheniformis* addition (Ramlucken et al., 2020). However, an improved body weight and FCR of 21–42 days and 1–42 days were seen in the PG and PA group although statistical difference was not reached, indicating that *B. licheniformis* and enramycin may protect broilers from SNE infection and alleviated growth performance loss caused by SNE to a certain degree. Studies had reported that probiotics *Bacillus* spp. increased the activity of digestive enzymes such as amylase and protease, and secreted some unknown growth-promoting factors which were helpful for intestinal development, feed degradation, and animal growth (Wang and Gu, 2010; Zhou et al., 2010). This may be one of the potential mechanisms by which probiotics *Bacillus* spp. improved the growth performance of SNE-infected broilers (Bai et al., 2016).

Intestinal lesion scores, intestinal microbiota balance, bacterial translocation, and intestinal morphology were important indicators for evaluating intestinal integrity and barrier function of broilers. The results of intestinal lesion scores in this study showed that the addition of *B. licheniformis* significantly reduced the duodenal and total intestinal lesion scores of SNE-infected broilers, which proved that the addition of *B. licheniformis* to diets effectively alleviated intestine damage caused by SNE infection in broilers. Consistent with our results, Wu et al. (2018) reported that *B. coagulans* significantly reduced intestinal lesion scores of broilers. The results from Jayaraman et al. (2013) also showed that the addition of *B. subtilis* PB6 significantly decreased the incidence and severity of intestinal lesions in *C. perfringens*-challenged broilers. In addition, in line with previous studies (Jayaraman et al., 2013; Wang H. et al., 2017; Wu et al., 2018), our results showed that SNE infection led to increased proliferation of cecal *C. perfringens* and higher *C. perfringens* invasion in the liver, indicating the imbalance of intestinal microflora and barrier damage of intestinal in broilers. After the supplementation of enramycin, lower *C. perfringens* load in the cecal contents and liver were observed compared with SNE-challenged birds, which proved that enramycin can effectively inhibit the growth of *C. perfringens*, thereby preventing pathogens or endotoxins from entering the systemic circulation. At 42 days of age, cecal *E. coli* numbers of broiler chickens in the antibiotics (enramycin) supplement group were significantly higher than that in the other groups. This may mainly be due to the growth inhibiting effect of enramycin on Gram-positive bacteria, especially to the harmful *Clostridium* in the intestinal tract, thus leading to the mass proliferation of Gram-negative bacteria such as *E. coli* (Sanjay et al., 2018). The intestinal mucus layer is the first defense barrier dialogued with microorganisms. The mucins secreted by goblet cells are important components of the mucus layer, providing a series of potential recognition sites for intestinal common microorganisms. At the same time, research noted that *C. perfringens* could not synthesize a variety of amino acids (Shimizu et al., 2002) so that the intestinal mucins served as an amino acid source of *C. perfringens* (Collier et al., 2008). In the present study, the addition of enramycin reduced the goblet cells in the jejunum, which may result in a decline in intestinal mucin secretion and a decreased availability of amino acids for *C. perfringens*, thereby inhibiting the growth and proliferation and translocation of *C. perfringens*. The morphological structure and integrity of intestine were associated with growth performance. Consistent with the growth performance that there was only numerically improvement between groups rather than differing significantly, the addition of *B. licheniformis* or enramycin had no significant improvement in the VH, CD, and VH/CD ratios in the jejunum of SNE-infected broilers. Inversely, Jayaraman et al. (2013) reported that diets added with *B. subtilis* PB6 significantly increased the intestinal VH and VH/CD ratio of NE-infected broilers, protected the intact villi structure of intestine, and reduced the FCR. As we know that the biofunction of probiotics varies from strain to strain, even the probiotic strains from the same species may work differently (Wang H. et al., 2017). Therefore, the contradictory results may partially be due to the different probiotic strains used in different studies. The aforementioned results also demonstrated that *B. licheniformis* and enramycin could mitigate intestinal injury of SNE-infected broilers in different aspects.

To determine the underlying mechanism responsible for this result, we further investigated the gene expression of tight junction proteins, *mucin-2* and TLR signaling pathway related factors, and the alterations in cecal microbiota. The intestinal tight junction (TJ) complex composed of occludins, claudins, ZOs, and other TJ proteins that control the intestinal paracellular permeability facilitates the exchange of water, ions, and other nutrients with external environment, and also plays an important role in resisting the invasion of intestinal pathogens and toxins (Awad et al., 2017). Many pathogens indirectly impair the TJ structures of the intestinal tract by activating the signaling cascades of the host cells (Wageha et al., 2017), while the *C. perfringens* enterotoxin directly uses the TJ proteins *Claudin-3* and *Claudin-4* as cellular receptors to attach, leading to TJ degradation and increased paracellular permeability (Fujita et al., 2000; Veshnyakova et al., 2010). In the present study, results showed that addition of *B. licheniformis* significantly down-regulated the expression of *Claudin-3* mRNA in the jejunum of SNE-infected broilers on day 25 and a decreasing trend was also observed in the enramycin group, indicating that *B. licheniformis* and enramycin can reduce the intestinal *C. perfringens* adhesion by down-regulating *Claudin-3* mRNA levels, thereby protecting the intestinal mechanical barrier and paracellular permeability. Mucin-2 is one of the mucins which participates in the formation of mucous layer and protects intestinal mucosal barrier integrity (Michael McGuckin et al., 2011). On day 42, *C. perfringens* loads in the cecal contents of SNE-infected broilers was decreased, and broilers in each group were in the late stage of SNE infection or in the normal health status as can be seen from the results of intestinal lesion scores. At this time point, the gene expressions of *Claudin-3* and *mucin-2* were significantly up-regulated in the enramycin supplemented group, and the mRNA levels of *mucin-2* were also increased in the *B. licheniformis* supplemented group, indicating that *B. licheniformis* and enramycin may convey a protection on the intestinal TJs and mucus layers of broilers (Aliakbarpour et al., 2012; Rajput et al., 2013). In addition, the results also showed that the addition of *B. licheniformis* or enramycin significantly increased the content of cecal formic acid in 42-day-old broilers. Formic acid, a kind of SCFA, is produced by intestinal bacteria fermenting undigested starch or fiber polysaccharides that are unused to the host. SCFAs have important physiological significance to the host (Meimandipour et al., 2010; Rinttila and Apajalahti, 2013), of which formic acid could improve the intestinal morphology of broilers (Garcia et al., 2007) and inhibit the growth of pathogens (Bourassa et al., 2018). The results of the present study showed that *B. licheniformis* or enramycin can maintain the intestinal health by increasing the content of cecal formic acid in SNE-infected broilers.

When intestinal pathogens invade the host, they can be recognized by pattern recognition receptors. For example, TLRs can identify various pathogen-related molecular patterns and transmit the signals downstream through their linker proteins such as *TRIF* and *MyD88* to activate *NF-κB*, which can be transferred into the nucleus and thus induce the expression of target genes to regulate the immune and inflammation response, cell proliferation, and regeneration (Wertz and Dixit, 2010; Huebener and Schwabe, 2013). In line with other studies (Jung et al., 2015; Rajput et al., 2017), we found that at the peak period of *C. perfringens* infection (2 DPI), adding *B. licheniformis* or enramycin to the diet significantly increased the jejunal mRNA expression of *TRIF* and *NF-κB* in broilers, which indicates the activation of TLR-NF-κB signaling pathway, although the mRNA levels of *TLR-4* and *TLR-2* were not changed. The TLR-NF-κB signaling pathway is included in the innate immune response. Its activation causes a series of signal transductions, which leads to the activation and cellular responses of immune-related cells, and subsequently induces the secretion of cytokines, growth factors (*TGF-β*, *IGF-2*, *GLP-2*), type I IFNs, and chemokines (Kawai and Akira, 2007). Cytokines are effector molecules that transmit information between immune cells and determine the nature of the immune response at the infection site. For example, *IL-17* secreted by Th17 cells is an inflammatory cytokine that stimulates the production of granulocytes, promotes the production of antimicrobial peptides by epithelial cells, and enhances innate immunity (Eyerich et al., 2017). Consistent with previous studies (Fasina and Lillehoj, 2018), in this experiment, the addition of enramycin significantly up-regulated the mRNA levels of *IL-17*, indicating that the innate immune function of the broiler intestines was enhanced and effectively resisted the *C. perfringens* infection. However, unlike previous results (Rajput et al., 2013, 2017; Wang Y. et al., 2017), our results found that *B. licheniformis* did not significantly affect the gene expression of cytokines, such as *IL-1β*, *IL-10*, *IL-17*, and *TNF-α* after activation of TLR-NF-κB signaling pathway. This may be due to the different physiological functions of different probiotic strains (Kang and Sin-Hyeog, 2015). Although there were no significant changes detected on the expression of cytokines, adding *B. licheniformis* significantly up-regulated the expression of growth factors (*GLP-2*, *TGF-β2*), *HSP60*, *HSP70*, and *HSP90*, which was consistent with previous studies (Selvam et al., 2009; Okamoto et al., 2012; Rajput et al., 2017; Tang et al., 2019). Growth factors can promote the cell differentiation, mucosal development, and repair of damaged tissues (Bulut et al., 2008; Massagué, 2012; Camati et al., 2017), while HSPs are anti-stress proteins with molecular chaperone activity that protects cells and tissues from temperature stress or protein denaturation caused by infection, enhancing the resistance to environmental stress (Lindquist and Craig, 1988; Malago et al., 2001). In this experiment, *B. licheniformis* activated the TLR-NF-κB signaling pathway in jejunum of SNE-infected broilers, and afterward up-regulating the expression of jejunal growth factors and HSPs, enhancing the ability of tissue repairing and anti-stress of host, but did not affect the gene expression of pro-inflammatory cytokines. Similarly, adding enramycin also activated the TLR-NF-κB signaling pathway in jejunum of 25-day-old SNE-infected broilers, and also increased the gene expression of growth factors (*IGF-2*, *GLP-2*, *TGF-β2*) and *HSP90*. Furthermore, the gene expression of the proinflammatory cytokine *IL-17* was also up-regulated in broilers. Therefore, *B. licheniformis* had an effect of enhancing immunity in contrast to the enramycin. However, the results on day 42 showed that enramycin down-regulated the TLR-NF-κB signaling pathway, the gene expression of pro-inflammatory cytokines and HSPs in jejunum of healthy broilers, indicating that enramycin decreased the level of immunity and anti-stress of broilers and thus transferred more energy and nutrients to animals for growing. Based on the aforementioned results, we suggested that the impact of *B. licheniformis* on broiler challenged with SNE was focused on the repair and anti-stress of intestine which was different from enramycin’s pro-inflammatory effects although they all activated the TLR-NF-κB signaling pathway.

Intestinal microbiota affects animal gut development, immune maturation, intestinal barrier, and host susceptibility to pathogens (Sekirov et al., 2010). Therefore, it is important to investigate infection, pre-treatment of *B. licheniformis*, or enramycin on intestinal microflora in broilers infected with SNE. Results showed that there was no significant difference in cecal microbiota α-diversity between groups. In accordance with our study, researchers found that α-diversity of gut microbiota was not affected by NE infection (Lin et al., 2017; Latorre et al., 2018), antibiotics supplementation (Sanjay et al., 2018), or probiotics *Bacillus* spp. (Qin et al., 2018) supplementation in broilers. We speculated that *C. perfringens* infection (Lin et al., 2017), and pre-treatment with antibiotics (Costa et al., 2017; Sanjay et al., 2018) or probiotics *Bacillus* spp. (Ahmed et al., 2014; Prieto et al., 2014) regulated the proliferation of minor microorganisms in the intestine of broilers thus the α-diversity in each group tended to be consistent. In terms of cecal bacterial community structure (β diversity), *B. licheniformis* adding group was similar to the enramycin group, but significantly different from the PC group, indicating that SNE infection caused a disturbance in cecal microflora in broilers, while adding *B. licheniformis* or enramycin modulated the bacterial community structure. Consistent with our results, Lin et al. (2017) and Xu et al. (2018) demonstrated that NE infection destroyed the community structure of intestinal microbes in broiler chickens and deviated it from normal state, whereas adding probiotics *Bacillus* spp. alleviated the disorder of intestinal microflora and restored it into homeostasis.

LEfSe analysis showed that the addition of enramycin reduced the relative abundance of *Peptostreptococcaceae*, *Intestinibacter*, and *Eisenbergiella* in SNE-infected broilers. It was reported that *Peptostreptococcaceae* were commensal bacteria in the intestine whose proportion in healthy animals was higher than that of diseased animals (Ma et al., 2011; Leng et al., 2016). However, other studies had also reported the presence of opportunistic pathogens in *Peptostreptococcaceae* may cause host disease (Ma et al., 2011). D’Andreano et al. (2017) compared the jejunal microflora of healthy and hemorrhagic enteritis turkeys, finding that *Peptostreptococcaceae* were only detected in the jejunum of hemorrhagic enteritis turkeys, which suggested that certain bacteria in *Peptostreptococcaceae* may also act as harmful bacteria and destroy the intestinal health of the host. For example, *Intestinibacter*, a genus of *Peptostreptococcaceae*, was significantly higher in the feces of patients with inflammatory diseases (such as Crohn’s disease) than that in healthy individuals (Forbes et al., 2018), which further confirmed our hypothesis. In addition, some studies suggested that the genus *Eisenbergiella* contains potential pathogenic bacteria (Bernard et al., 2017). Bao et al. (2018) reported that the abundance of *Eisenbergiella* was significantly increased in the feces of *Echinococcus granulosus*-infected rats, and speculated that *Eisenbergiella* might be associated with the host’s Th2 immune response. Therefore, in the present study, the enhanced intestinal barrier function and decreased *C. perfringens* liver translocation of broilers in enramycin group may partially relate to the decreased abundance of *Peptostreptococcaceae*, *Intestinibacter*, and *Eisenbergiella*. Compared with the PC group, we noted that the addition of *B. licheniformis* increased the abundance of *Lachnospiraceae_UCG_010* in the cecum of SNE-infected broilers. Researchers reported that the abundance of *Lachnospiraceae_UCG_010* was significantly reduced in feces of patients with irritable bowel syndrome, while it was increased in healthy individuals (Zhuang et al., 2018), suggesting that *Lachnospiraceae_UCG_010* may be beneficial intestinal bacteria and positively correlate with intestinal health. In this experiment, the increased abundance of *Lachnospiraceae_UCG_010* in the *B. licheniformis* supplement group was consistent with an increase in intestinal barrier function and a decline in intestinal lesion scores. In line with our results, many studies reported that probiotics *Bacillus* spp. modified the intestinal microflora of NE-infected broilers (Langille et al., 2013; Lin et al., 2017; Xu et al., 2018). Moreover, *Clostridiales_vadinBB60_group* and one of its genera were enriched in the *B. licheniformis* group when compared with the enramycin group. *Clostridiales_vadinBB60_group* contains a variety of bacteria producing butyric acid. Studies had reported that the increased abundance of *Clostridiales_vadinBB60_group* was accompanied by the enhanced serum antioxidant capacity in mice (Shimizu et al., 2002). In addition, Zhang et al. (2018) noted that the presence of *Clostridiales_vadinBB60_group* was detected in the feces of diabetic rats after treated with liraglutide. It was speculated that *Clostridiales_vadinBB60_group* may also be beneficial bacteria in intestinal tract, which was good for host health. *Ruminococcaceae_NK4A214_group* may also be a potentially beneficial bacterium. Studies had demonstrated that the abundance of *Ruminococcaceae_NK4A214_group* was reduced in obese rats and gout patients (Shao et al., 2017; Zhao et al., 2017). In contrast, *Family_XIII_AD3011_group* is considered to be a potential pathogen, and many studies reported that the abundance of *Family_XIII_AD3011_group* is positively associated with the diabetes (Zhang et al., 2018). Our results showed that the addition of *B. licheniformis* increased the abundance of *Clostridiales_vadinBB60_group*, *g_uncultured_bacterium_f_Clostridiales_vadinBB60_group*, *Family_XIII_AD3011_group*, and *Ruminococcaceae_NK4A214_ group* were enriched in *B. licheniformis* supplemented group compared with the enramycin group, indicating that the recovery effect of *B. licheniformis* on cecal microbiome disorders of SNE-infected broilers is better than enramycin.

The observed shifts in the intestinal microbiota may regulate gut physiological function, host health, and growth. The PICRUSt aims to predict the unobserved characters from phylogenetic information regarding the organisms in the community. Vitamins are organic compounds which could be produced by bacteria notably vitamin K and B groups, regulating the construction and supporting normal physiological function of host (Rowland et al., 2018). An important role it serves is being cofactors for enzymes. Results presented the metabolism of cofactors and vitamins pathway was enriched in group PG, indicating a positive regulative effect of *B. licheniformis* on the activity of enzymatic metabolism (Rozs et al., 2001). However, it was lower in group PA which may result from the antibiotic effect of enramycin. Membrane transport, a vital pathway for the survival of bacteria in the gut ecosystem (Lyons et al., 2017), was increased in group PA, which may point out an attempt to compensate for the antibiotic effect of enramycin. Regarding energy metabolism pathway, it was enriched in group PC comparing with group PG and PA, showing an energy metabolites disorder in SNE infection broilers (Chan et al., 2020). Cell motility is the determinant step of pathogen bacteria in early local invasion (Lin et al., 2017). The abundance of cell motility was enriched in PC group as compared with the PA and PG group, indicating the anti-infective effect of *B. licheniformis* and enramycin. In addition, it was found that carbohydrate metabolism pathways were enriched in group PA and PG, according with increased concentration of formic acid in cecum content. As reported, carbohydrate could be metabolized by microflora into SCFAs which were known to boost intestinal health by its trophic and anti-inflammatory effects (Kles and Chang, 2006; Lamas et al., 2018). Qing et al. (2018) noted that SNE infection could affect the hepatic lipid metabolism of chickens and probiotic pretreatment may provide a prophylaxis strategy against SNE infection through regulating lipid metabolism (Zhou et al., 2016; Lin et al., 2017). Agreed with those reports, we found that *B. licheniformis* supplement down-regulated the abundance of lipid metabolism pathway; however, enramycin up-regulated it, showing the different regulative effect of *B. licheniformis* and enramycin on cecum microbial function in the SNE-challenged broilers. Amino acid metabolite polyamines such as putrescine, spermidine, and spermine are harmful to hosts (Stevanato et al., 2012; Bonaiuto et al., 2015). Nevertheless, it was revealed that polyamines support gut physiology by strengthening barrier function, promoting gut maturation, increasing anti-oxidant capacity, and regulating immune function (Lagishetty and Naik, 2008; Bekebrede et al., 2020). As shown that amino acid metabolism pathway was enriched in group PG, this may confirm the hypothesis that *B. licheniformis* could adjust immune function through activating TLR-NF-κB signaling pathway. Therefore, the effect of *B. licheniformis* or enramycin on SNE-challenged broilers needs to be further investigated. Thus, dietary supplementation with antibiotic enramycin and probiotic *B. licheniformis* affected important predicted functions of the intestinal microbiota of the NE-challenged birds.

## Conclusion

Dietary supplementation with *B. licheniformis* or enramycin mitigated the negative effects of SNE infection in broilers and alleviated intestinal damage, suggesting *B. licheniformis* could be used as an antibiotic alternative. *B. licheniformis* protected the intestinal health of SNE-infected broilers mainly mediated by increasing the number of beneficial bacteria *Lachnospiraceae_UCG_010* and formate acid content in the cecum, modulating TLR-NF-κB signaling pathway, and up-regulating jejunal *mucin-2*, growth factor (*GLP-2* and *TGF-β2*), and HSP (*HSP60*, *HSP70*, and *HSP90*) mRNA levels. However, the addition of enramycin maintained the intestinal barrier function mediated by reducing intestinal and liver *C. perfringens* load, increasing the cecal formate acid concentration, affecting the TLR-NF-κB signaling pathway, and up-regulating intestinal tight junction protein *Claudin-3*, *mucin-2*, pro-inflammatory cytokines together with growth factors and HSPs. This study showed that there are similarities and differences on the mechanism of *B. licheniformis* and enramycin in relieving intestinal damage of SNE-infected broilers. More studies are needed to confirm these results in the future.

## Data Availability Statement

The original contributions presented in the study are publicly available. This data can be found here: https://www.ncbi.nlm.nih.gov/, PRJNA728387.

## Ethics Statement

The animal study was reviewed and approved by China Agricultural University Animal Care and Use Committee (statement no. CAU20170601-2).

## Author Contributions

ZW designed the research. LK, YL, and VP performed the experiments and analyzed the data. FG wrote the manuscript. ZW and YG participated in the revision of the manuscript. All authors contributed to data interpretation and approved the final version of the article.

## Conflict of Interest

The authors declare that the research was conducted in the absence of any commercial or financial relationships that could be construed as a potential conflict of interest.

## Footnotes

1 http://huttenhower.sph.harvard.edu/lefse/
