# Supplementary material for: Probiotics Bacillus licheniformis Improves Intestinal Health of Subclinical Necrotic Enteritis-Challenged Broilers
Source: Front Microbiol. 2021 May 18;12:623739. doi: 10.3389/fmicb.2021.623739 (PMC8168541; doi:10.3389/fmicb.2021.623739)
Supplement: Supplementary file 1 [file Data_Sheet_1.docx]

Supplementary Material

# Supplementary Figures


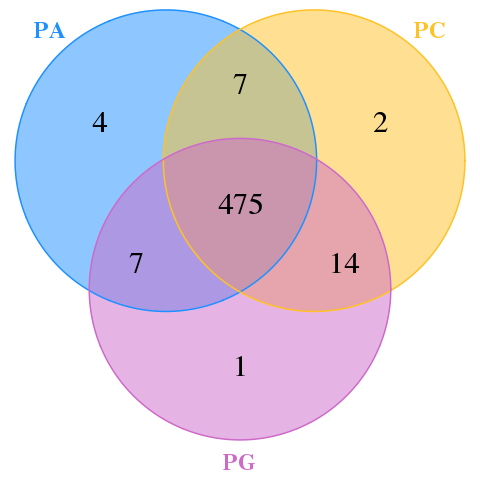


**Figure S1.** Venn diagram of OTUs in different groups. PC: basal diet + SNE; PA: basal diet extra antibiotics + SNE; PG: basal diet extra Gallipro Tect^®^ + SNE.

A B


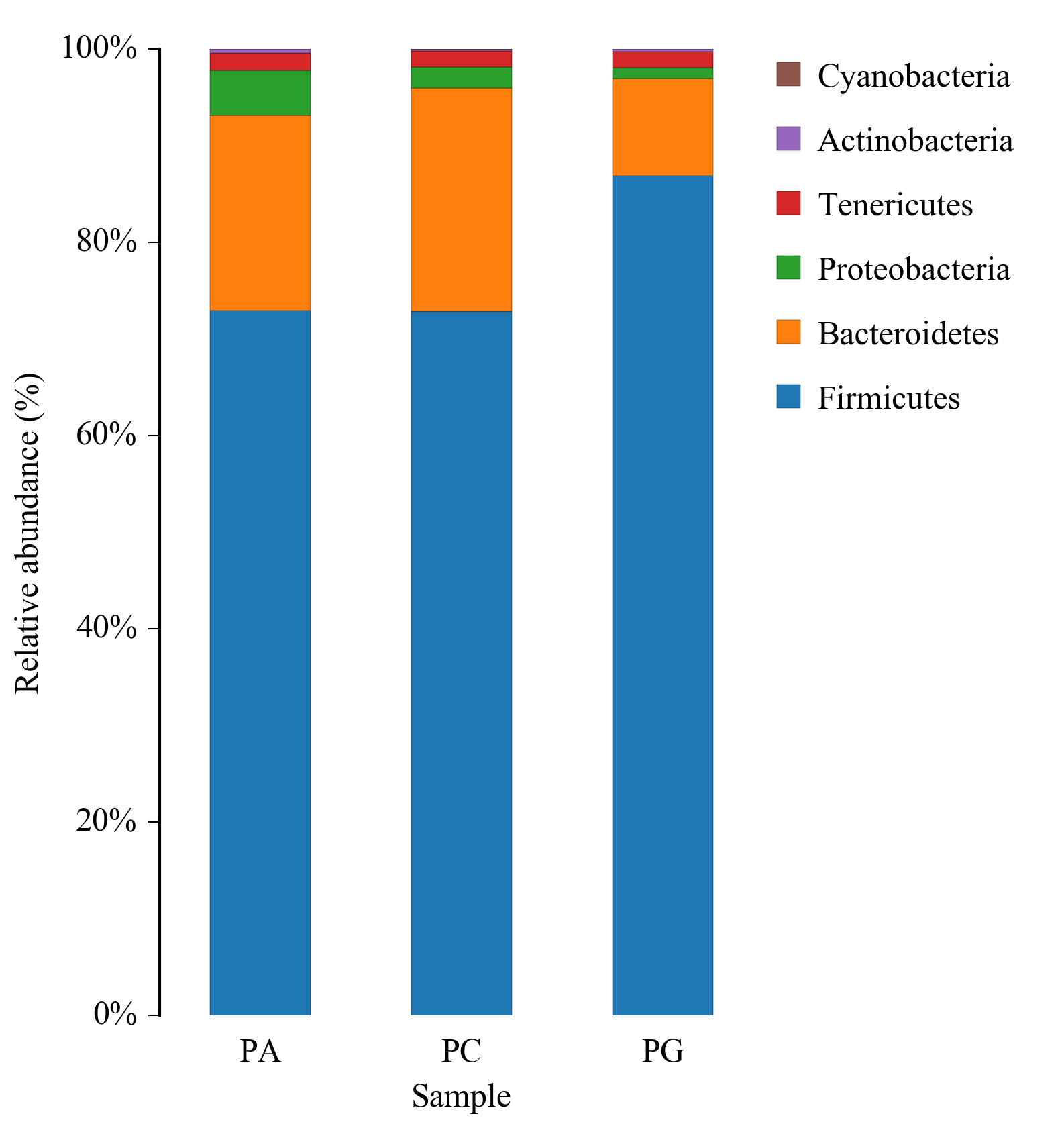

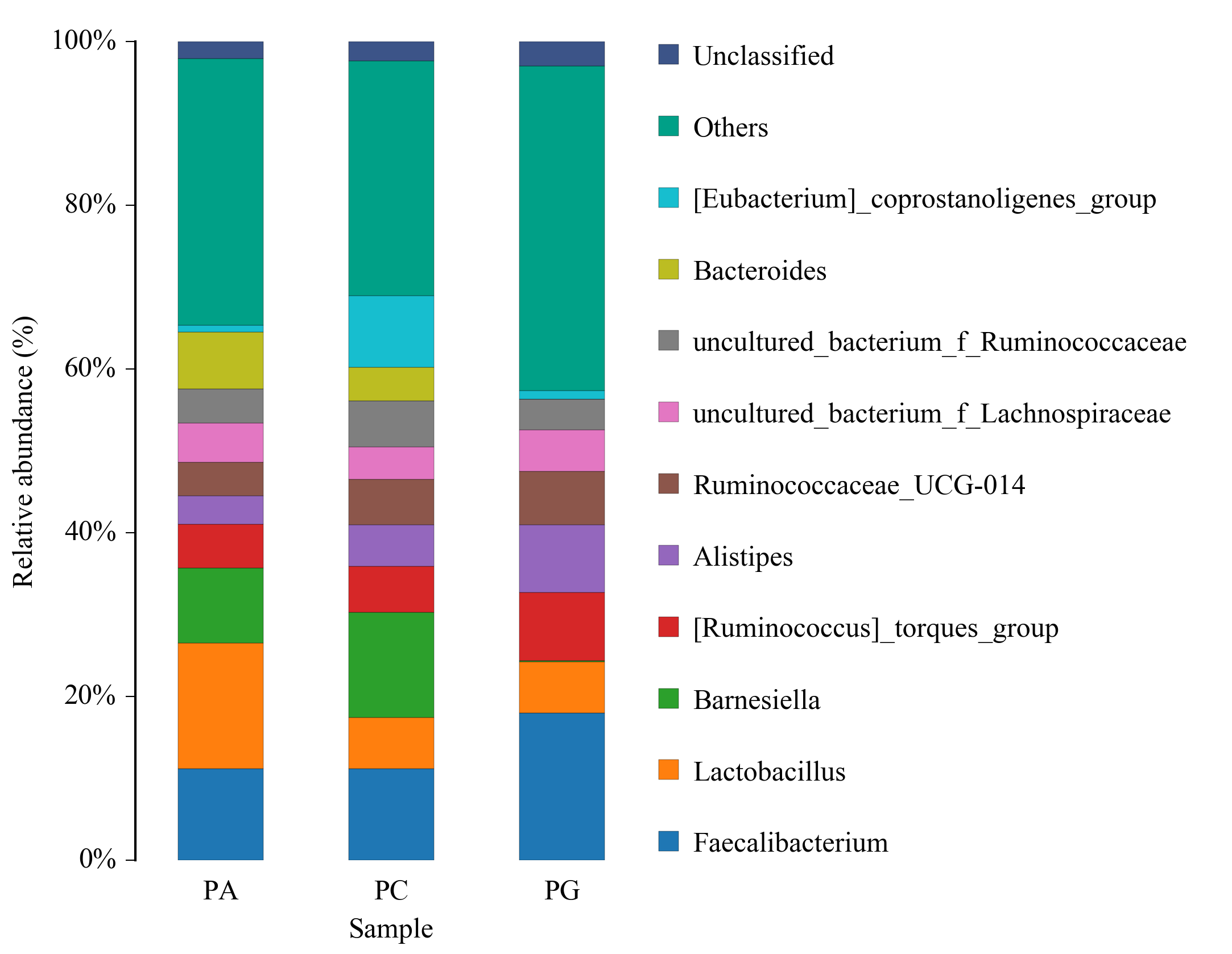


**Figure S2.** Effects of *B. licheniformis* and enramycin on bacterial relative abundance at phylum **(A)** and genus levels **(B)** in cecal microbiota of 25-day-old broilers infected with SNE. PC: basal diet + SNE; PA: basal diet extra antibiotics + SNE; PG: basal diet extra Gallipro Tect^®^ + SNE.

# Supplementary Tables

**Table S1.** Primer sequences of RT-PCR

| **Target genes** | **Primer sequence (5'~3')** | **Accession NO.** |  |
| --- | --- | --- | --- |
| β-actin | F：GAGAAATTGTGCGTGACATCA  R：CCTGAACCTCTCATTGCCA | L08165 |  |
| GAPDH | F：GGTGGTGCTAAGCGTGTTAT  R：ACCTCTGTCATCTCTCCACA | K01485 |  |
| Occludin | F：ACGGCAGCACCTACCTCAA  R：GGGCGAAGAAGCAGA`TGAG | NM-205128.1 |  |
| Claudin-1 | F：CATACTCCTGGGTCTGGTTGGT  R：GACAGCCATCCGCATCTTCT | AY750897.1 |  |
| Claudin-3 | F：GCTCTGCCGTTACCAGCTACG  R：CTGCACACAGCTCTCCTGGCAAC | NM_204202.1 |  |
| ZO-1 | F：CTTCAGGTGTTTCTCTTCCTCCTC  R：CTGTGGTTTCATGGCTGGATC | XM_413773 |  |
| Mucin-2 | F：TTCATGATGCCTGCTCTTGTG  R：CCTGAGCCTTGGTACATTCTTGT | XM_421035 |  |
| TLR-4 | F：GGATCTTTCAAGGTGCCACA  R：CAAGTGTCCGATGGGTAGGT | AY064697 |  |
| TLR-2 | F：CTGGGAAGTGGATTGTGGA  R：AAGGCGAAAGTGCGAGAAA | AB050005.2 |  |
| TRIF | F：TCAGCCATTCTCCGTCCTCTTCAG  R：GGCACAGGAAGTTCTTGGTCAGCA | NM_001081506.1 | |
| MyD88 | F：TGCAAGACCATGAAGAACGA  R：TCACGGCAGCAAGAGAGATT | NM_001030962.3 | |
| NF-κB | F：GTGTGAAGAAACGGGAACTG  R：GGCACGGTTGTCATAGATGG | NM_205129.1 | |
| IL-1β | F：ACTGGGCATCAAGGGCTA  R：GGTAGAAGATGAAGCGGGTC | XM_015297469.1 |  |
| IL-10 | F：CGGGAGCTGAGGGTGAA  R：GTGAAGAAGCGGTGACAGC | EF554720.1 |  |
| IL-17 | F：AGCTGACGGTGGACCTATTATT  R：GGCTTTGCGCTGGATTC | NM_205149.1 |  |
| IFN-γ | F：CATGGTACCTGTGGCAATACC  R：GCACTGAGCGGATTACTTCC | NM_001006471 |  |
| TNF-α | F：GAGCGTTGACTTGGCTGTC  R：AAGCAACAACCAGCTATGCAC | XM_204267 |  |

*F, forward; R, reverse. Primers were synthesized by Sango Biotech (Shanghai) Co., Ltd.*

**Table S2. Primer sequences of RT-PCR**

| **Target genes** | **Primer sequence (5'~3')** | **Accession no.** |
| --- | --- | --- |
| GAPDH | F：GGTGGTGCTAAGCGTGTTAT  R：ACCTCTGTCATCTCTCCACA | K01485 |
| HSP60 | F：GGTGATGCTTGCAGTTGATG  R：TTGCCAATTTCCTGATCTCC | [NM_001012916.2](https://www.ncbi.nlm.nih.gov/nuccore/NM_001012916.2) |
| HSP70 | F：CCACCTACGCAAAGAGGAAG  R：TGAGGTGTTGGGTTCCTTTC | [NM_](https://www.ncbi.nlm.nih.gov/nuccore/NM_001006685.1)001030793.2 |
| HSP90 | F：TGAGGACGTTGGTTCTGATG  R：TGGTCCAAATAGGCTTGGTC | [NM_001109785.1](https://www.ncbi.nlm.nih.gov/nuccore/NM_001109785.1) |
| IGF-2 | F：TGGCTCTGCTGGAAACCTAC  R：ACTTGGCATGAGATGGCTTC | XM_015286525.2 |
| GLP-2 | F：AAGCTTCCCAGTCTGAACCA  R：ATCCTGAGCTCGTCTGCTGT | NM_001190165.4 |
| TGF-β2 | F：TCATCACCAGGACAGCGTTA  R：TGTGATGGAGCCATTCATGT | NM_001031045.3 |

*F, forward; R, reverse: Primers were synthesized by Sango Biotech (Shanghai) Co., Ltd.*
